# Deep temporal nerve transfer: A systematic review of anatomy, anatomical landmarks and clinical applications

**DOI:** 10.1016/j.jpra.2022.05.007

**Published:** 2022-05-21

**Authors:** Frank W. de Jongh, Sjaak Pouwels, Elijah E. Sanches, Niels van Heerbeek, Koen J.A.O. Ingels

**Affiliations:** a Department of Otorhinolaryngology, Head & Neck Surgery, Radboud University Medical Center, Nijmegen, The Netherlands; b Department of Intensive Care Medicine, Elisabeth-Tweesteden Hospital, Tilburg, the Netherlands

**Keywords:** Deep temporal nerve transfer, Nervus temporalis profundus, Facial palsy, Facial reanimation surgery, Plastic surgery, Anatomy

## Abstract

**Background:**

Facial paralysis has a debilitating impact on facial function, especially the eyebrow. Static procedures have long been the standard treatment strategy, but in more recent years, dynamic procedures are being developed. To reconstruct the upper branches of the facial nerve (temporal and zygomatic branches), the deep temporal nerve (DTN) and its branches, with its close proximity to the eyebrow, can be used as a possible donor nerve for reinnervation. This systematic review provides an overview of the DTN anatomy and possible surgical treatment strategies.

**Materials and methods:**

A PubMed systematic literature search was performed in October 2021. Studies on cadavers and clinical studies providing anatomical or surgical information on the dissection of the DTN as well as surgical treatment strategies using the DTN were included.

**Results:**

The literature search produced 311 results, including 36 duplicates. After screening on title and abstract, 11 studies were found possibly relevant and underwent a full-text critical appraisal, resulting in 4 exclusions. In total seven studies were included. The data gathered were used to adequately describe the DTN anatomy, surgical approaches and landmarks used during surgery.

**Conclusion:**

The DTN and its branches prove to be a viable donor for the reconstruction of facial nerve branches, since the location and anatomical consistency can be relied upon through a multitude of studies, especially of the middle branch. Our study describes anatomy and nerve characteristics for its use in facial reanimation.

## Introduction

Since the beginning of the 20th century, the temporalis muscle (TM) is used in facial reconstructive surgery, in particular, in dynamic reconstructions of the face in patients with a peripheral facial palsy (PFP). ^1^The main goals of facial reanimation are to decrease the hyper functionality of the face, improve symmetry and balance the paralyzed area of the face by improving the resting tone and the emotive facial movement, e.g., smiling.^2^ The effects of facial paralysis are often far reaching, like oral incompetence, cosmetic depreciation, speech difficulties and the inability to express emotions, leading to significant psychosocial problems.^2^

Sir Harold Gillies was the first to reconstruct deformities caused by the loss of the zygoma bone, with TM flaps.^3^ The use of the temporalis flap has grown over the last century and is now often used for the closure of maxillary defects after tumour resections, as interpositional material to treat temporomandibular joint ankylosis and for a dynamic muscle transfer for patients with a PFP.^3^ Gillies's work was improved by McLaughlin by mobilising the insertion of the temporalis instead of the origin. Labbé modified this technique further in the 1990s, reporting a temporalis lengthening without using an interposed fascial graft.^1^ Despite the fact that the Labbé technique has several advantages, the main disadvantage is that there is no spontaneous movement possible in the reconstructed area.^1^ Dynamic smile restoration is one of the current goals of (dynamic) facial reanimation.^2^

Currently, facial reanimation focuses on dynamic techniques using nerve transfers. According to Banks et al., facial nerve transfers are possible when the following criteria are met: a proximal facial nerve that is unavailable for repair, intact distal nerve branches and receptive facial musculature.^2^ The following nerves are commonly used to restore facial movement and tone: the motor branches of the trigeminal nerve V3, the facial nerve (VII), the spinal accessory nerve (XI), the hypoglossal nerve (XII) and the cervical rootlets.^2^ Deep temporal-to-facial nerve transfers (V-to-VII nerve transfer) are more frequently used in the facial reanimation surgery due to the fact that it provides reliable and rapid reinnervation, low functional morbidity, and in most cases, does not require a nerve graft due to the small distance to the facial nerve branches.^2^.

The knowledge of the innervation in the area of the TM is still not fully clear. The deep temporal nerve (DTN) could play an important role in spontaneous movement after facial reanimation surgery. The goal of this review is to give an overview of the current literature on facial reanimation after facial palsy using the DTNs and their clinical application.

## Methods

A multi-database search (in PubMed, Medline, Embase and The Cochrane Library) was conducted between the earliest dates of each database until 27 October 2021. Our specific interest was the surgical anatomy of the DTN, in particular, strategies on how to locate the nerve, related anatomical landmarks and its anatomical properties (e.g., branches, length of the DTN trunk and distances between the DTN and anatomical landmarks). This study was performed and reported in accordance with the Preferred Reporting Items for Systematic Reviews and Meta-Analyses (PRISMA) guidelines.^3^^,^^4^

The search was conducted using medical subject headings (MeSH) and a combination of keywords from the following two groups: (a) “facial palsy”, “peripheral facial palsy” and “facial plastic surgery”; (b) “deep temporal nerve”, “temporal nerve”, “dynamic reconstruction” and “smile reanimation surgery.”

Authors FdJ and SP individually screened and selected studies on the basis of title and abstract. After primary selection, the authors reviewed the full text of the selected studies and determined their suitability for inclusion. For further eligible studies, cross-references were screened. Disagreements were solved by a discussion with each other, and the senior author (KI) until consensus was reached.

For cumulative quantitative synthesis, we only included full-length published studies that reported experience with the anatomy of the DTN in either cadaveric or animal or clinical studies. We excluded studies with only abstracts, review articles and clinical practice guidelines. Articles written in Dutch, English, German and French were included.

## Data extraction and statistical analysis

Data from each of the studies retrieved include type of study (clinical, cadaveric or animal study), numbers of dissections, anatomical landmarks related to the DTN, aspects of the DTN (branches, distances to anatomical landmarks) and indications for harvesting (branches of) the DTN. In case of consistent and homogeneous data reporting among the included studies, a meta-analysis will be done using the DerSimonian and Laird random-effects model.^5^ In all tests, values of p<0.05 were considered statistically significant. Statistical Package for Social Sciences (SPSS, Chicago, IL, USA Version 20.0) was used to prepare a database and for statistical analysis.

## Results

The primary literature search produced 311 results, including 36 duplicates. After screening on title and abstract, 11 studies were found possibly relevant and underwent a full-text critical appraisal, resulting in 4 exclusions. Reasons for exclusion were the following: one survey study, two reviews and one anatomical study that did not report anatomical landmarks. In total, 7 studies were included in this systematic review.^2^^,^^6^, ^7^, ^8^, ^9^, ^10^, ^11^

**Figure 1:**
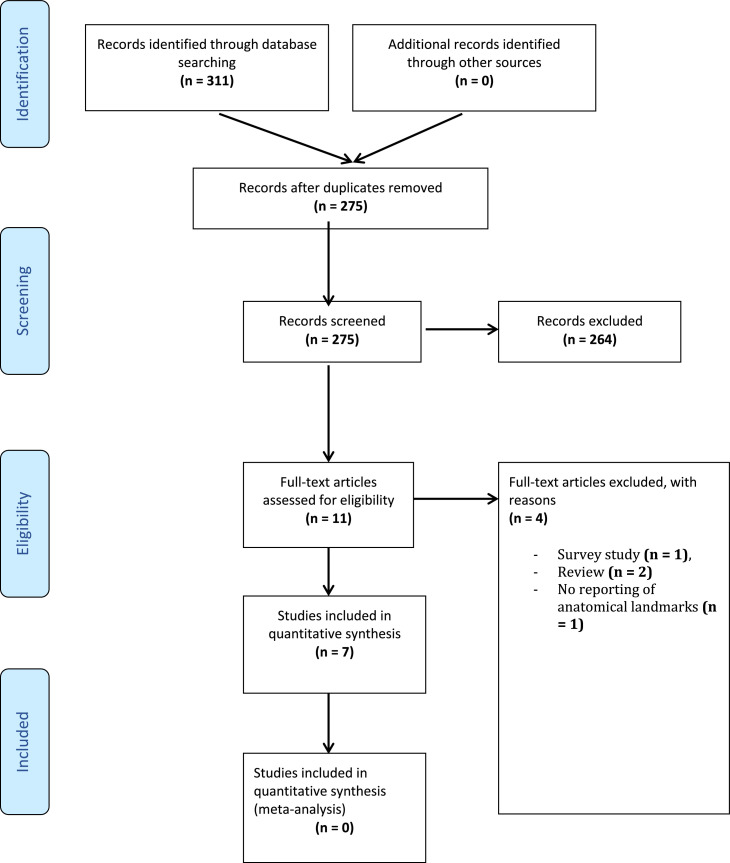
PRISMA flowchart.

Figure 1 summarises the search results, according to Preferred Reporting Items for Systematic Reviews and Meta-Analyses (PRISMA) guidelines.^3^^,^^4^ Table 1 gives an overview of the results of the included studies. Due to significant heterogeneity in reported data, a meta-analysis was not performed.

**Table 1:** Study characteristics

Study	Cadaveric/clinical	Patients (m/f)	Number of dissections (uni-/bi-lateral)	Investigated	Landmarks used	Number of DTN branches (mean (range))
Ali A 2012	Cadaveric	11 (5/6)	17 (5/6)	Examining DTN in number, branching pattern, length and diameter	IFT, PDTA, ITC	3.24^2^, ^3^, ^4^, ^5^
Banks CA 2019	Clinical	5 (x/x)	5 (5/0)	Experiences with V to VII nerve transfers for smile reanimation	TM, ZA	x
Karagoz H 2015	Cadaveric	5 (2/3)	10 (0/5)	Evaluating the anatomy of the anterior, middle and posterior deep temporal nerves; nerves to the temporalis muscle; and their availability for direct muscle neurotisation of the orbicularis oculi	IFT, ZA, TM	2.8^2^, ^3^ (anterior and posterior branches were found in all specimens)
Kwak HH 2003	Cadaveric	22 (22/14)	36 (X/X)	Clarifying the positional relationships and the clinical relevance of the course variations of the branches of the anterior mandibular nerve trunk with reference to the surrounding anatomical structures	IFT, LPMs	X
Mahan MA 2016	Cadaveric + case report	8 (x/x) + 1(1/0)	16 (0/8)	Surgical approach to DTN for nVII reconstruction. Distance of DTN branch to jugal point	JP	3^3^ out of 3 bilateral dissections
Dauwe PB 2016	Cadaveric	16 (7/9)	30 (0/15) + 6 (0/3) surgical approaches	Surgical approach to DTN for nVII reconstruction. Distance of DTN branches to tragus and zygomatic arch	ZA, Tragus	2.47^2^, ^3^ (23/30 anterior, 30/30 middle, 20/30 posterior)
Staples 2021	Cadaveric	2 (x/x)	4 (0/2) preserved + 4 (0/2) unpreserved	Surgical approach DTN transfer for eyelid reconstruction	ZA, Tragus	3^3^ out of 4 preserved facial halves

Abbreviations: DTN: deep temporal nerve, IFT: infratemporal fossa, ITC: infratemporal crest, PDTA: posterior deep temporal artery, ITF: infratemporal crest, ZA: zygomatic arch, TM: temporalis muscle, LPMs: superior head of lateral pterygoid muscle, JP: jugal point

### Study characteristics

Of the included studies, five were cadaveric studies^6^, ^7^, ^8^, ^9^^,^^11^, one clinical^2^ and one a combination of a cadaveric study and a case report^10^. The studies did a total of 123 dissections on 66 cadavers^6^, ^7^, ^8^, ^9^^,^^11^ (of which 2 unpreserved dissections^11^) and 6 clinical operations.^2^^,^^10^ Landmarks used were the infratemporal fossa (IFT),^7^, ^8^, ^9^ infratemporal crest (ITC),^7^ posterior deep temporal artery (PDTA),^7^ zygomatic arch (ZA),^3^^,^^6^^,^^8^^,^^11^ TM,^3^^,^^8^ superior head of lateral pterygoid muscle (LPM),^9^ jugal point (JP)^10^ and the tragus.^6^^,^^11^

### Anatomy

The DTNs are motor branches of the anterior division of the mandibular division (nV3) of the trigeminal nerve (nV). The posterior DTN (PDTN) forms a common trunk with the masseteric nerve, and DTN trunk length was found to be 7.19 mm (SD 2.04) and diameter of 0.93 mm (SD 0.30).^7^ The DTNs divide of the anterior division and course over or through the LPM and ascending to the temporal fossa deep to the TM.^9^ The length from the bifurcation of the DTN to the muscle insertion is 6.99 mm (SD 4.12).^7^ The further course of the DTN nerve differs greatly where several branching types have been observed.^7^ Ali et al. measured the diameter of the DTN trunk (0.93 mm (SD 0.30). Diameters of the different branches were also measured by Ali et al., who found up to five branches ranging from 0.26–0.52 mm (no location division was noted), while Karagoz et al. found an anterior branch diameter of 0.54 mm (SD 0.12), a middle branch diameter of 0.50 mm (SD 0.16) and a posterior branch diameter of 0.49 mm (0.18).^7^

The number of DTN branches found was mentioned in 5 studies with a mean 2.82 and range 2–5, and Ali et al. was the only author reporting four or five branches^6^, ^7^, ^8^^,^^10^, ^11^, ^12^. Ali et al. also reported different branching patterns, where 35% (6/17 cadavers) depicting an almost equal division of the DTN trunk when emerging from the IFT. Also, a further sub-branching of the posterior branch (35%) or the anterior branch (29%) was observed. Dauwe et al. found that the middle branch was most consistent in appearance and size (30/30 middle, 23/30 anterior and 20/30 posterior).^6^

Kwak et al. studied the passing of the PDTN in origin and branching patterns. They found that the LPM was not only innervated by the buccal nerve but also the anterior and middle DTN branches. Also, in several cases, the middle DTN (MDTN) could originate from the masseteric nerve or the PDTN.^9^

### Landmarks and surgical approach

Ali et al. used the IFT, the PDTA and the ITC to localise, measure and elucidate the DTN nerves to provide the basis for advances in facial reanimation techniques. They found a fairly consistent entry point in the muscle of 71% in the middle third of the deep/posterior surface. This is also the location of crossover of the PDTA related to the DTN.^7^

The distance between the DTN origin in the ITC and terminal muscle entry points was 46.4 mm (range 42–51) for the anterior branch, 42.2 mm (range 38–46) for the middle and 33.4 mm (range 26–40) for the posterior branch as measured by Karagoz et al.^8^

In the article by Karagoz et al. the anterior deep temporalis nerve (ADTN) was investigated, and to approach the ADTN, the temporal muscle, fascia and ZA were exposed after which the TM was dissected in the plane created from the superior aspect exposing the DTN. After detaching the TM, the length of the ADTN was assessed for orbicularis oculi muscle neurotisation.^8^

Dauwe et al. conducted a study to assess which branch is most feasible for functional muscle and nerve transfers in this specific area. After the dissection of 30 hemifaces, establishing the anatomy in this area and performing axonal analysis, the authors concluded that the middle branch was the most viable for transfers, for both nerves and functional muscle, due to its highest rate of appearance and its high axon count (1469 axons at ZA level).^6^

Banks et al. describe a preauricular incision, which is extended superiorly into the temporal region and 2 cm inferior to the mandible angle. The TM is dissected from the bone posterior to anterior, and the DTN branches are identified and isolated on the underside of the muscle.^2^ The authors used the DTN to reconstruct facial nerve branches when the reinnervation potential was discussed to leave the masseteric nerve open for gracilis reconstruction if it were to fail, three out of five DTN-to-facial nerve transfers were successful (versus the 88% success rate of the masseteric nerve reconstructions).^2^

In the study by Mahan et al., a reliable surgical approach to the MDTN was devised the feasibility of transferring it to the upper division of the facial nerve for reconstruction, utilising this nerve transfer on a single facial palsy patient.^10^ They found the ADTN within -5 to + 5 mm of the JP at the superior zygoma border and the main trunks of the MDTN and PDTN, respectively, between 9 and 15 mm and 17 and 28 mm posterior to the JP at the superior zygoma border.^10^

Staples et al. measured the location of the DTN branches from the most posterior aspect of the tragus at the superior border of the ZA. The anterior branch was found at 5.5 cm (SD 0.6), the middle branch at 4.6 cm (SD 0.7) and the posterior branch at 2.8 cm (0.2). They also found that the MDTN was most consistent in location and course, suggesting a planned incision at 4.6 cm anterior to the most posterior point of the tragus along the superior border of the ZA.^11^

## Discussion

This article describes the anatomy of the DTN and its branches in full as it is known in the current literature available and provides information about the surgical approach and which landmarks to use to plan for the reconstruction of affected facial nerve branches.

The classic representation of the innervation of the TM by the DTN is as follows: the anterior (ADTN), MDTN, and PDTN innervate the temporal muscle.^12^ These DTNs originate from the anterior trunk of the mandibular branch of the trigeminal nerve.^12^ Besides the earlier mentioned pattern, the ADT nerve originates from the buccal nerve, and the PDT nerve originates from the masseteric nerve.^12^

Ali et al. mention the anterior and posterior branches, further branching in a maximum of five total DTN branches, while Dauwe et al. mention the middle branch as most consistent nerve between maximum three possible branches. It is unclear whether the middle branch as described by Dauwe et al. is consistent with the posterior branch as described by Ali et al.^6^^,^^7^

Banks et al. mostly used masseteric-to-facial nerve transfers, and only used the DTN to preserve the masseteric nerve in patients with concerns about possible reinnervation to keep the option for gracilis transfer open if the nerve transfer were to fail. In the five DTN-to-facial nerve transfers, three were successful.^2^ The authors suggest the DTN as an excellent option for nerve transfer since there is minimal donor site morbidity, and the selection and reconstruction can occur within the same surgical wound.^2^

The middle branch could serve as a potential donor nerve for eyebrow or periorbital reanimation surgery.^6^^,^^11^ The surgical approach used to expose the middle branch of the DTN is as follows:^6^ to expose the deep temporal fascia, a preauricular incision is prolonged into the scalp to become a hemicoronal incision.^6^ The surgical plane then exposes the supraorbital rim and ZA.^6^ The earlier measurements of this study of the middle and anterior division of the DTN were used to mark the starting points for fascia incision and to subsequently bluntly dissect the muscle fibres to find the nerves.^6^ After the nerves are identified, they could be dissected from the muscle fibres surrounding it.^6^

## Limitations

The main limitation of this review is the variability between the included studies. All studies explore different approaches, landmarks and DTN (branch) characteristics. Because of this, it is not possible to objectively compare results or draw clear conclusions regarding the optimal approach or the best landmarks used during surgery.

## Conclusion

The DTN and its branches are suggested to be a viable donor for the reconstruction of facial nerve branches, since the location and anatomical consistency can be relied upon through a multitude of studies, especially of the middle branch. This approach is already used in practice, although more clinical research is needed to further cement the DTN as a possible donor in the reconstruction toolbox for facial reanimation for patients afflicted by facial paralysis.

## Author Contributions

**Initial idea:** SP, KI

**Literature search:** FdJ, SP, ES

**Writing the article:** FdJ, SP, ES, NvH, KI

**Final approval:** FdJ, SP, ES, NvH, KI

## Declaration of Competing Interest

None.

## Funding

None.

## Ethical Approval

Not required.
